# Hematologic response assessment in monoclonal gammopathy of renal significance: critical appraisal of current criteria

**DOI:** 10.3389/fmed.2026.1902202

**Published:** 2026-09-08

**Authors:** A. Domingo-González, P. Cannata-Ortiz, R. Fernández-Prado, A. Velasco-Valdazo, M. Menéndez-Cuevas, R. Martos, L. Pavía Pascual, E. Prieto Pareja, D. Naya, B. García Corral, I. Mahíllo-Fernández, P. Llamas Sillero, L. Solán, E. Askari

**Affiliations:** 1 Department of Hematology, Fundación Jiménez Díaz University Hospital, Madrid, Spain; 2 Health Research Institute, IIS-Fundación Jiménez Díaz, Madrid, Spain; 3 Department of Pathology, Fundación Jiménez Díaz University Hospital, Madrid, Spain; 4 Department of Nephrology, Fundación Jiménez Díaz University Hospital, Madrid, Spain; 5 Department of Hematology, Rey Juan Carlos University Hospital, Madrid, Spain; 6 Department of Hematology, General de Villalba Hospital, Madrid, Spain; 7 Department of Hematology, Infanta Elena Hospital, Madrid, Spain; 8 Department of Clinical Analysis, Fundación Jiménez Díaz University Hospital, Madrid, Spain; 9 Division of Statistics and Epidemiology, Fundación Jiménez Díaz University Hospital, Madrid, Spain

**Keywords:** monoclonal gammopathy of renal significance, MGRS, MGRS response assessment, MGRS hematologic evaluation, MGRS renal evaluation, MGRS measurable disease

## Abstract

**Introduction:**

Current recommendations for hematologic response (HemR) assessment in monoclonal gammopathies of renal significance (MGRS) are based on the monoclonal immunoglobulin (MIg) component responsible for renal damage. Light-chain (LC) amyloidosis criteria are recommended when renal lesions are due to LC, whereas multiple myeloma (MM) criteria are proposed when due to an intact monoclonal immunoglobulin. However, the nephrotoxic MIg is frequently not measurable either by the International Myeloma Working Group (IMWG) or the International Society of Amyloidosis (ISA) criteria.

**Methods:**

In the study shown below, we retrospectively analyzed an original multi-center series of 45 MGRS patients.

**Results:**

Thirty-two MGRS-non-amyloidosis (MGRS-NA) and 13 MGRS-amyloidosis (MGRS-A) patients were included. Twelve (37.5%) and 15 (47%) MGRS-NA patients had measurable disease by either the IMWG or ISA criteria, respectively. According to the IMWG criteria, 5% of MGRS-NA patients with intact MIg renal deposits and 80% with exclusively LC deposits had measurable disease, as determined by serum electrophoresis or sFLC assay, respectively. Based on the ISA criteria, 35 and 80% of MGRS-NA patients with MIg and LC renal deposits, respectively, had measurable disease.

**Discussion:**

Our findings reveal the limitations of the current proposed HemR assessment in MGRS-NA patients and explore potential alternatives to ease clinical practice and guideline development.

## Introduction

Monoclonal gammopathy of renal significance (MGRS) is defined as a clonal population of plasma cells or B lymphocytes that produces a nephrotoxic complete or partial monoclonal immunoglobulin (MIg) and does not meet any other criteria for treatment other than the renal injury caused by the MIg’s physicochemical properties (1–3). Despite recent advances in diagnosis and histological classification, management guidelines for MGRS other than light chain (LC) amyloidosis have not yet been established (4–6), and prospective trials have barely been conducted (7, 8). Recommendations on behalf of the International Kidney and Monoclonal Gammopathy Research Group (IKMG) encourage measuring hematologic response (HemR) based on the MIg component involved in kidney injury. In MGRS patients, the International Society of Amyloidosis (ISA) response criteria are proposed when renal damage is caused by monoclonal LC, whereas the International Myeloma Working Group (IMWG) criteria are suggested when renal damage involves an intact Ig (5). However, the criteria used in real life are heterogeneous, and do not consider the MIg causing renal damage (7, 9). This study aims to identify the limitations of current recommendations and explore alternative assessments for HemR in MGRS-NA.

## Patients and methods

### Study population

This multi-center, retrospective, and observational study included patients diagnosed with MGRS between January 2016 and January 2025. Patients were recruited from four hospitals, all sharing the same pathologist. Inclusion criteria required the determination of serum MIg isotype, serum free LC (sFLC) measurement, serum monoclonal spike (M-spike) by electrophoresis, and renal biopsy at diagnosis. Urine M-spike was not mandatory due to missing data. Patients with symptomatic MM, CLL, or NHL diagnoses were excluded. Patients were identified through the pathology registry. The records, including demographics and disease characteristics, were retrospectively collected from the electronic medical records.

### Monoclonal protein measurements

Serum and urine M-protein were quantified by serum and urine electrophoresis (sPEP/uPEP). The serum FLC assay was performed using ® FREELITE (The Binding Site, Birmingham, United Kingdom).

### Bone marrow evaluation

The percentage of bone marrow (BM) plasmacytosis and lymphocytosis was determined by manual cell counting from BM aspiration, while monoclonality was assessed by flow cytometry. When the BM biopsy was available, this percentage was determined by immunohistochemistry.

### Renal histology

Formalin fixed and paraffin embedded renal biopsies from the four hospitals were evaluated by light microscopy, immunofluorescence, both direct immunofluorescence on frozen tissue and on paraffin embedded tissue after enzymatic digestion, and electron microscopy in the same pathology department. The following histopathological features were assessed: number of glomeruli, presence or absence of proliferative lesions (and their typification), global and/or segmental glomerulosclerosis, deposits, state of glomerular basement membrane, acute tubular damage, interstitial fibrosis, interstitial inflammation, and tubular atrophy. Immunofluorescence staining was graded from 0 to 3 + for LC (kappa and lambda), immunoglobulins (IgG, IgA, IgM), and complement factors (C1q, C3), as well as fibrinogen and albumin. Cases with histological findings incompatible with MGRS diagnosis, such as cast nephropathy or neoplastic infiltration, were excluded.

### Hematologic response evaluation

Hematologic response was assessed according to two established guidelines: the IMWG criteria for MM (10) and the updated ISA criteria for AL amyloidosis (11, 12). Overall HemR was defined as achieving at least a partial response. Bone marrow evaluation was not required to define a complete response. Supplementary Tables S1, S2 show response categories based on the IMWG criteria and updated ISA criteria, respectively. Measurable disease according to the IMWG was determined as serum M-protein ≥1 g/dL by SPEP, urine M-protein ≥200 mg/24 h by UPEP, and involved sFLC level ≥100 mg/L, provided sFLC ratio is abnormal by FLC assay (10). According to the IMWG criteria, in the absence of measurable disease at baseline, the HemR can only be defined as a binary response (complete response or progressive disease). Measurable disease according to the ISA was defined as a difference between involved and uninvolved FLC > 50 mg/L, provided detectable disease. Detectable disease was defined as positive immunofixation in serum or urine. According to the ISA criteria, when the difference between the involved and uninvolved FLC is between 20 and 50 mg/L, the HemR should be ungraded (complete response, non-complete response, or progressive disease). Measurable disease and HemR were only assessed in patients with detectable disease. Hematologic response was performed at the end of each treatment cycle.

### Renal response evaluation

Renal response (RenalR) was assessed by proteinuria in accordance to the organ response criteria for AL amyloidosis (11), which include the following categories: complete response (CR) defined by a nadir proteinuria ≤200 mg/day, very good partial response (VGPR) >60% reduction in proteinuria to nadir value >200 mg/day, partial response (PR) 31–60% reduction in proteinuria, stable disease (SD) ≤ 30% reduction in proteinuria. Proteinuria was considered measurable if ≥500 mg/day at diagnosis. Since AL amyloidosis response criteria do not include renal progression definition, it was defined according to the physician’s criteria.

### Statistical methods

Qualitative variables were summarized as counts and percentages and compared by the Chi-squared test or Fisher’s exact test when the assumptions for the Chi-squared test were not met. Quantitative variables were summarized as median and interquartile range (IQR), and compared using the Mann–Whitney *U* test. All reported *p*-values are two-sided and were considered significant if less than 0.05. All statistical analyses were performed using R (R Core Team, 2025), version 4.4.2 (R Foundation for Statistical Computing, Vienna, Austria).

### Ethics approval and consent to participate

All methods were performed in accordance with the relevant guidelines and regulations. The study was approved by the IIS-Fundación Jiménez Díaz institutional ethics committee and conducted in accordance with the 1964 Declaration of Helsinki and its later amendments (EO057-24_FJD).

## Results

### Patient characteristics

**Figure 1:**
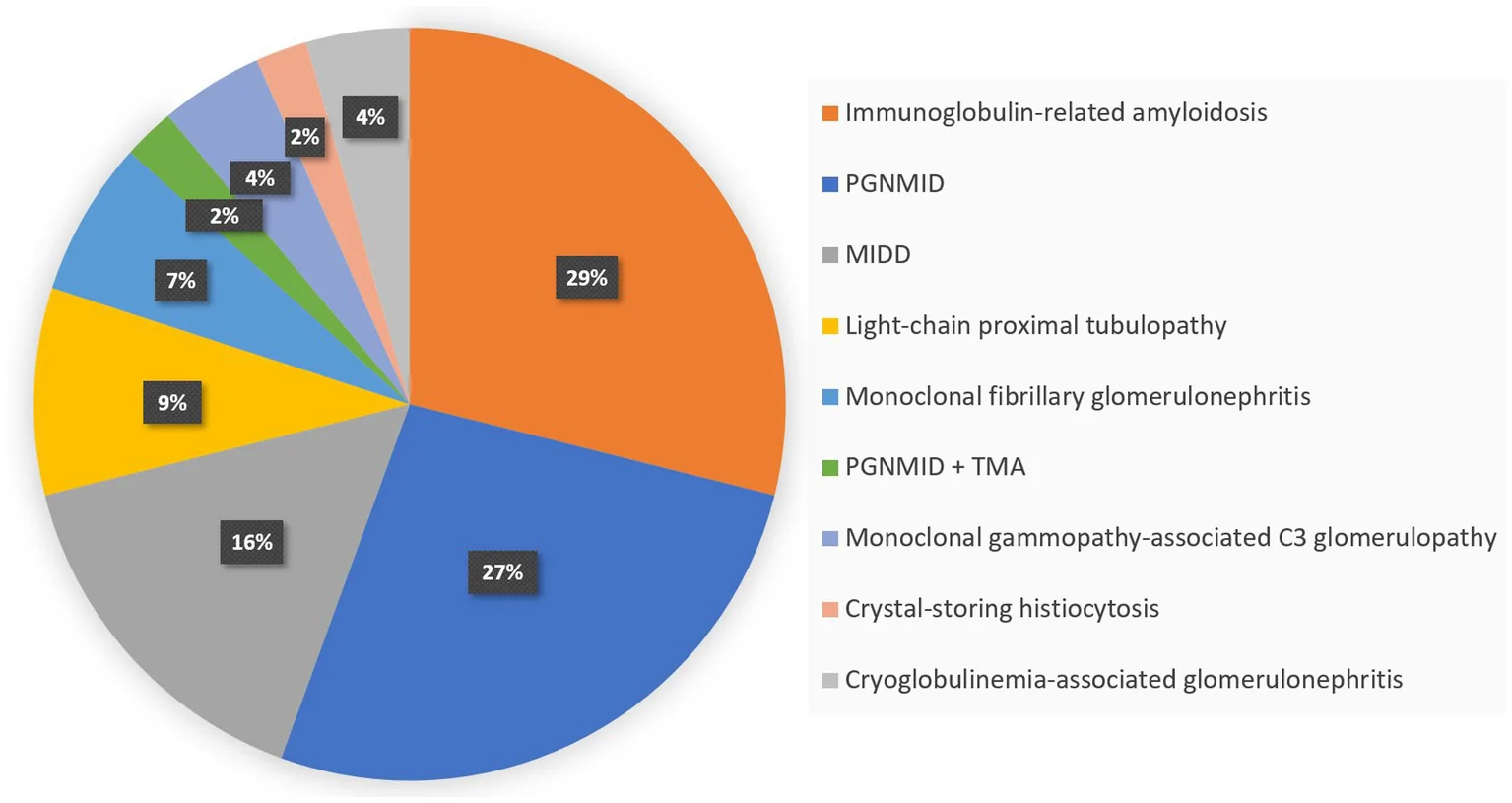
Distribution according to MGRS type. MIDD: Monoclonal immunoglobulin deposition disease, PGNMID: Proliferative glomerulonephritis with monoclonal Ig deposition, TMA: Thrombotic microangiopathy.

Forty-five patients diagnosed with MGRS had a renal biopsy and a hematologic evaluation, including MIg isotype, levels of sFLC, and serum M-spike measurement at diagnosis. Eight (18%) patients had no available uPEP at baseline. Thirteen (29%) had MGRS consisting of LC amyloidosis (MGRS-A), and 32 (71%) had MGRS-non-amyloidosis (MGRS-NA). Figure 1 shows the distribution by MGRS type. Light-chain amyloidosis was the most frequent MGRS, followed by proliferative glomerulonephritis with MIg deposition (PGNMID), and MIg deposition disease (MIDD).

Forty (89%) and 23 (51%) patients underwent BM evaluation and whole-body imaging, respectively. Twelve (27%) patients met the criteria for smoldering MM, one (2%) for concomitant smoldering MM and CLL, and one (2%) for asymptomatic CLL. In 22 (49%) cases, monoclonal cells were identified in BM evaluation, but the criteria for hematologic neoplasia were not met. Additionally, 9 (20%) cases had undetectable monoclonal cells, and all were in the MGRS-NA group. The median age at diagnosis was 66 years (interquartile range (IQR) 52–72), and 28 (62%) patients were male. Table 1 shows baseline patient characteristics at diagnosis according to MGRS type (MGRS-A vs. MGRS-NA).

**Table 1:** Baseline patient characteristics at diagnosis according to MGRS type.

Clinical features			
Total no.	MGRS-A *N* = 13	MGRS-NA *N* = 32	*p* value
Age (years), median (IQR)	69 (61–71)	63 (52–72)	0.270
Female/male, distribution—no. (%)	6 (46%)/7 (54%)	11 (34%)/21 (66%)	0.511
Laboratory characteristics			
	Median (IQR)	Median (IQR)	*p* value
Hemoglobin (serum)—g/dL	13.1 (12.2–13.7)	11.8 (10.2–13.9)	0.042
Creatinine (serum)—mg/dL	1.3 (0.8–2.0)	1.4 (1.1–2.8)	0.276
Glomerular Filtration—mL/min/m^2^	42 (26.1–91.0)	43 (24.0–58.2)	0.374
Protein (urine)—mg/24 h	3,717 (2814–10,500)	3,123 (651–5,084)	0.082
Albumin (urine)—mg/24 h	2,876 (2022–6,702)	2,503 (365–3,746)	0.031
Detectable MP (serum or urine)—distribution—no. (%)	12 (92%)	23 (72%)	0.238
LC restriction (serum or urine)—Kappa/Lambda—no. (%)	1 (8%)/11 (92%)	14 (61%)/9 (39%)	0.004
MP by proteinogram (serum) g/dL if detectable—Median (IQR)	0.3 (0.2–0.4)	0.3 (0.1–0.8)	0.608
MP by proteinogram (urine) g/dL if available—Median (IQR)	0.4 (0.17–1.0)	0.05 (0–0.22)	0.003
Total no.	*N* = 8	*N* = 13	
Involved FLC level mg/L provided serum FLC ratio is abnormal—Median (IQR)	160 (113–201)	484 (272–1,190)	0.009
Total no.	*N* = 12	*N* = 23	
Difference between involved and uninvolved FLC mg/L if DD—Median (IQR)	111 (71.9–177)	107 (19.0–488)	1.000
Bone marrow			
Total no.	*N* = 13	*N* = 27	*p* value
Monoclonal cells, distribution—no. (%) - Plasma cells < 10%/plasma cells ≥ 10% - Lymphocytes - No detectable clonality	7 (54%)/6 (46%) 0 (0%) 0 (0%)	8 (28%)/9 (31%) 3 (11%) 8 (30%)	0.168
Type of clonality, distribution—no. (%) - Kappa/Lambda	2 (15%)/11 (85%)	11 (58%)/8 (42%)	0.028
Kidney biopsy			
Total no.	*N* = 13	*N* = 32	*p* value
Median number of glomeruli, median (IQR)	30 (24–37)	25.5 (18.8–39.2)	0.831
Glomeruloesclerosis—%, median (IQR)	6.2 (4–11.1)	11 (3.7–26.2)	0.498
Tubular atrophy and/or interstitial fibrosis >25%, distribution—no. (%)	2 (15%)	5 (15%)	1.000
Exclusively LC renal deposition, distribution—no. (%)	8 (62%)	10 (32%)	0.094
Immunofluorescence staining Kappa/Lambda, Distribution—no. (%)	2 (25%)/6 (75%)	5 (50%)/5 (50%)	0.366
MIg renal deposition, distribution—no. (%)	5 (38%)	20 (63%)	0.191
Immunofluorescence staining IgG/IgA/IgM, distribution—no. (%)	4 (80%)/0 (0%)/1 (20%)	16 (80%)/1 (5%)/7 (35%)	1.000
Immunofluorescence staining Kappa/Lambda, distribution—no. (%)	0 (0%)/5 (100%)	18 (90%)/2 (10%)	<0.001

DD: Detectable disease, LC: Light chain, MIg: Monoclonal immunoglobulin, MP: Monoclonal protein, sPEP: Serum protein electrophoresis, uPEP: Urine protein electrophoresis. ^1^Only 11 MGRS-A patients and 26 MGRS-NA patients had uPEP available.

Seventeen (53%) MGRS-NA patients had measurable disease by the IMWG and/or ISA criteria. There were 12 (37.5%) and 15 (47%) cases with measurable disease according to the IMWG and ISA criteria, respectively. Among MGRS-A patients, 11 (85%) had measurable disease, 9 (70%) by the IMWG criteria, and 10 (77%) by the ISA. All MGRS cases with unmeasurable disease had a baseline BM plasma-cell ≤ 30%. The median follow-up was 39 months (IQR 18–62) and 43 months (IQR 23–80) in the MGRS-NA and MGRS-A group, respectively.

## MGRS-NA patients

### Renal histology

Ten (31%) MGRS-NA patients had exclusively LC renal deposition, 20 (63%) had an entire MIg deposition, and 2 (6%) had no M-protein renal deposition. Among patients with MIg renal deposition, 4 (20%) had predominant LC deposition by immunofluorescence staining. We found significant discrepancies between the M-protein detected in renal histology and the one identified by immunofixation in serum and/or urine, since 44% of the patients with a complete Ig in serum/urine had only LC renal deposition.

### Measurable disease by the IMWG

Three (15%) patients with MGRS-NA due to complete MIg renal deposition had measurable disease by the IMWG criteria. Of those, one (5%) had measurable disease by sPEP/uPEP/FLC assay, one (5%) by uPEP, and one (5%) by sFLC assay. Of ten patients with MGRS-NA due to exclusive LC renal deposition, 80% had measurable disease by FLC assay, and the remaining 20% had no measurable disease by either sPEP or uPEP.

### Measurable disease by the ISA criteria

Seven (35%) patients with MGRS-NA due to a complete MIg renal deposition had measurable disease by ISA, whereas eight (80%) patients with exclusive LC renal deposition had measurable disease.

**Figure 2:**
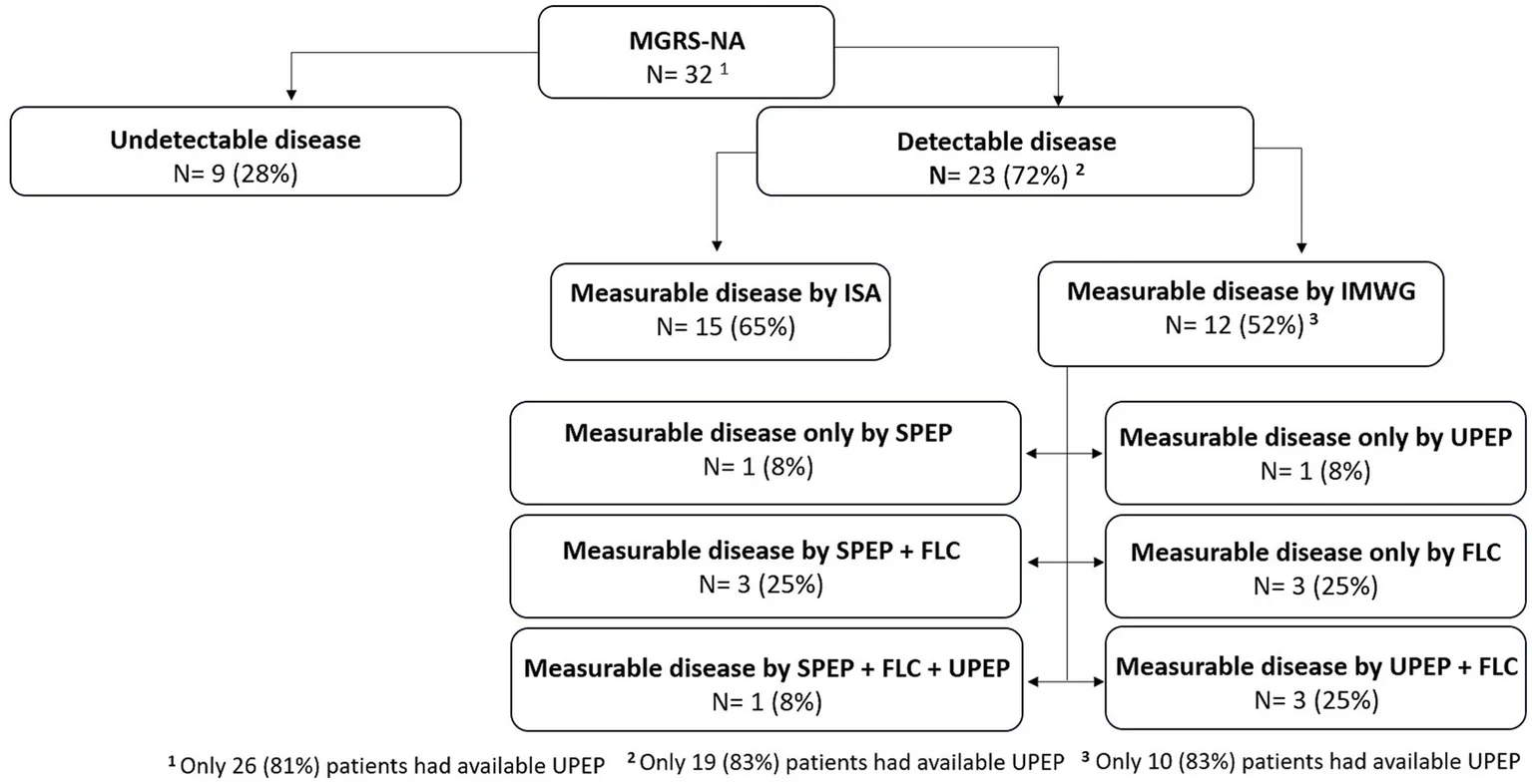
Detectable and measurable disease in MGRS-NA patients. FLC: Free light chain, IMWG: International Myeloma Working Group, ISA: International Society of Amyloidosis, SPEP: Serum protein electrophoresis, UPEP: Urine protein electrophoresis.

Figure 2 shows detectable and measurable disease in MGRS-NA patients.

## MGRS-A patients

### Renal histology

All MGRS-A patients had exclusively or predominantly LC renal deposition (62 and 38%, respectively).

### Measurable disease by the IMWG criteria

No patient had measurable disease by sPEP. Nine (70%) MGRS-A patients had measurable disease by IMWG, 90% by FLC, and 10% only by uPEP.

### Measurable disease by the ISA criteria

Ten (77%) patients had measurable disease by the ISA criteria.

**Figure 3:**
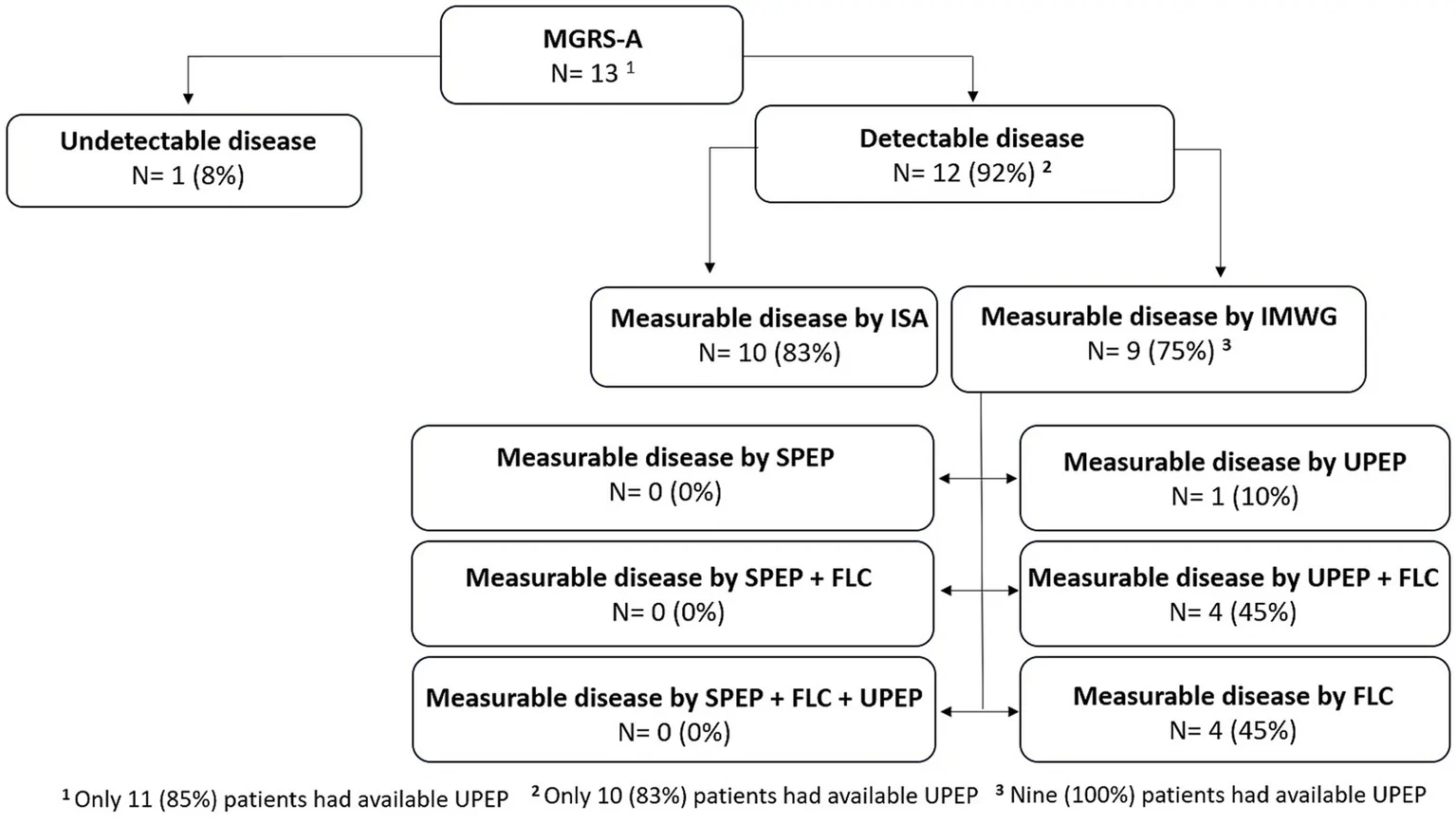
Detectable and measurable disease in MGRS-A patients. FLC: free light chain, IMWG: International Myeloma Working Group, ISA: International Society of Amyloidosis, SPEP: serum protein electrophoresis, UPEP: urine protein electrophoresis.

Figure 3 represents detectable and measurable disease in MGRS-A patients.

## MGRS-NA patients

### Treatment and response

Twenty-seven (85%) patients received hematologic treatment. Two PGNMID, one light-chain proximal tubulopathy, one monoclonal fibrillary glomerulonephritis, and one monoclonal gammopathy-associated C3 glomerulopathy did not receive hematologic therapy. In 24 (90%) patients, treatment targeted monoclonal plasma cells, whereas in 3 (10%) cases it was directed at monoclonal lymphocytes. Sixteen (60%) received a double/triplet regimen based on bortezomib, four (15%) a triplet/quadruplet based on daratumumab, three (10%) steroids plus an alkylating agent, three (10%) rituximab, and 1 (5%) steroids plus an immunomodulator agent.

#### MGRS-NA hematologic response (HemR)

The overall HemR rate among patients with measurable disease was 85% by IMWG criteria and 80% by ISA criteria. All responders’ patients achieved at least a VGPR by ISA and 90% by IMWG criteria. Ten (37%) patients had measurable disease by both criteria, and all of them achieved the same best HemR.

#### MGRS-NA renal response (RenalR)

Twenty-three patients (72%) received hematologic treatment and had evaluable renal assessments. The overall RenalR rate was 70% (10% PR, 40% VGPR, and 20% CR). Five patients (20%) had renal progression. Three patients required renal replacement therapy, two temporarily and one permanently.

Ninety percent of MGRS-NA patients who achieved a HemR (by ISA or IMWG) achieved a RenalR, whereas 65 and 100% of those who did not (by ISA and IMWG, respectively) could not reach a RenalR (*p* = 0.108 and *p* = 0.222).

## MGRS-A patients

### Treatment and response

Thirteen (100%) patients received hematologic treatment to eliminate monoclonal plasma cells. Eight (62%) received a double/triplet regimen based on bortezomib, four (30%) a triplet/quadruplet based on daratumumab, and one (8%) steroids plus an alkylating agent.

#### MGRS-A hematologic response (HemR)

The overall HemR rate among those patients with measurable disease was 100% by the ISA and 90% by the IMWG. Every responder patient achieved at least VGPR regardless of the criteria used. When disease was measurable by both criteria, there were no discrepancies in the classification of the best HemR.

#### MGRS-A renal response (RenalR)

**Figure 4:**
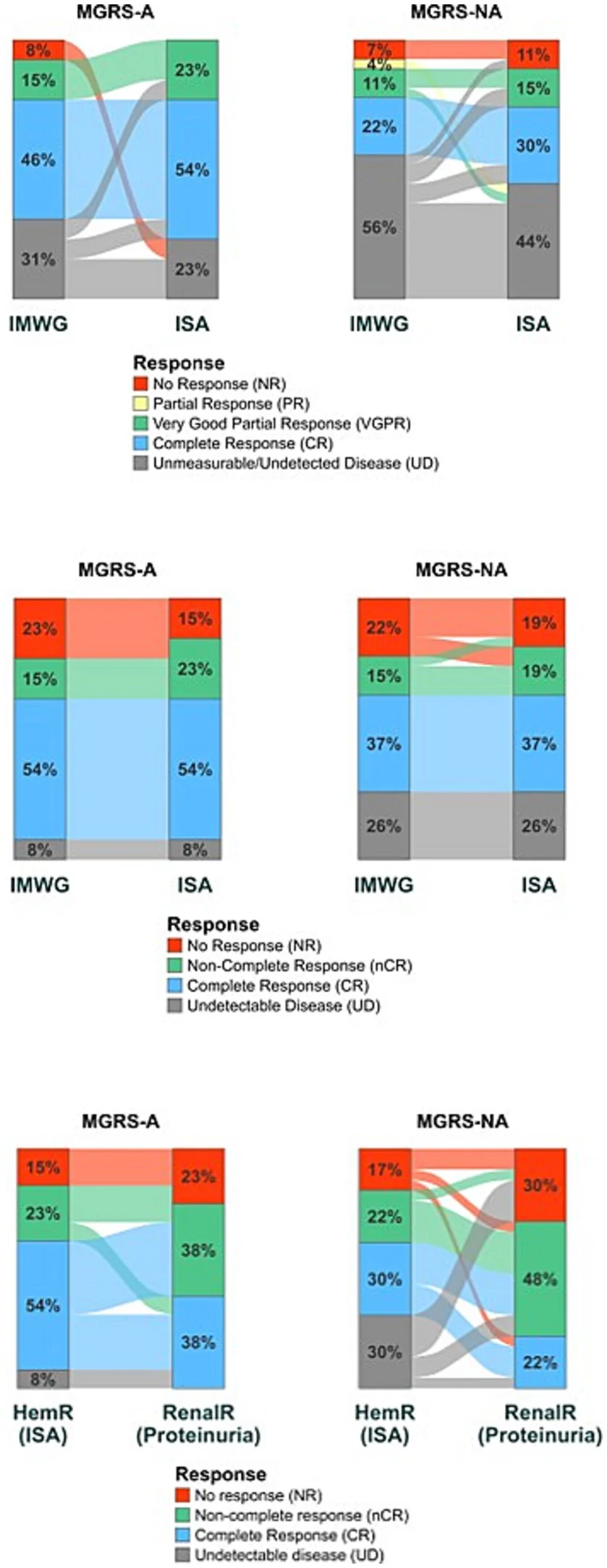
Panel **(A)** displays the best HemR achieved in MGRS-NA and MGRS-A patients according to the IMWG and ISA criteria. Panel **(B)** shows HemR according to disease measurability status: ungraded (complete response/non-complete response/no response) when measurable; or binary (complete response/no response) when unmeasurable. Panel **(C)** displays the best RenalR achieved in MGRS-NA and MGRS-A patients according to the best HemR (ungraded/binary).

Thirteen patients (100%) received hematologic treatment and had RenalR assessed. The overall RenalR rate was 77% (7% PR, 30% VGPR, and 40% CR). Two patients (16%) achieved stable disease, and one patient progressed (7%), ending up on permanent renal replacement therapy Figure 4.

Ninety percent of MGRS-A patients who achieved a HemR (by ISA or IMWG) achieved a RenalR.

Regarding the proposed simplified HemR (ungraded/binary), 90% of MGRS-A patients who achieved a HemR showed a RenalR, whereas those with no HemR did not reach a RenalR (*p* = 0.045). In the MGRS-NA group, 90% of patients with a HemR obtained a RenalR, compared to 50% of those without a HemR (*p* = 0.121).

## Discussion

The current recommendations for HemR assessment in MGRS-NA have certain limitations. Because of the disease’s heterogeneity and rarity, real-world criteria do not consider MIg-mediated renal damage (7, 9). Furthermore, our results show that, due to the disease’s low burden, HemR assessment cannot rely on the MIg component identified in renal histology.

This work highlights three fundamental questions:

1. Could a uniform HemR evaluation be applied to all patients?
2. Which HemR assessment provides greater applicability: the IMWG or the updated ISA criteria?
3. Considering the small clone size of MGRS-NA patients, could we simplify the HemR assessment?
4. Could a uniform HemR evaluation be applied to all patients?

The results of our study show a close association between the HemR and the RenalR, regardless of the criteria used. However, only 17 (53%) MGRS-NA patients had measurable disease by the IMWG and/or ISA criteria. When an intact MIg causes renal damage, it is frequently not measurable by sPEP according to the IMWG criteria (9). This entails response evaluation based on uPEP, which is infrequently performed in real life (13, 14), or sFLC levels, mimicking the criteria in AL amyloidosis (11, 12). In our study, only one (5%) patient with MGRS-NA due to complete MIg renal deposition had measurable disease by sPEP, and it was also measurable by uPEP/sFLC assay. On the other hand, 8 (80%) patients with MGRS-NA caused by exclusively LC had measurable disease by sFLC assay, and the remaining 20% had no quantifiable disease by either sPEP or uPEP. Regarding the ISA criteria, 35% of MGRS-NA patients with a complete MIg renal deposition had measurable disease, compared to 80% of MGRS-NA patients with exclusive LC renal deposition. These results reveal that HemR evaluation cannot depend on the MIg component identified in renal histology, which raises two questions. Should we use the sFLC assay to evaluate HemR, regardless of the renal M-protein? Should we stop assessing the HemR if MGRS-NA is caused by a complete MIg?

2 Which HemR assessment provides greater applicability: the IMWG or the updated ISA criteria?

In our series, 28% of MGRS-NA patients had no detectable M-protein in serum and/or urine. Therefore, HemR could not be assessed in approximately one-third of patients. For the rest, the ISA criteria enabled the assessment of HemR in a larger proportion of patients (65%) compared to the IMWG criteria (52%). Additionally, if the HemR evaluation was based exclusively on sFLC, the ISA criteria were even superior to the IMWG (65% vs. 44%). This is especially important in MGRS-NA patients with a complete MIg renal deposition, since only 15% of them had measurable disease by the IMWG criteria as opposed to 35% when using the ISA criteria. On the other hand, when evaluating the best HemR achieved in MGRS patients with measurable disease, there was 100% agreement, irrespective of the criteria used.

3 Considering the small clone size of MGRS-NA patients, could we simplify the HemR assessment?

Among MGRS-NA patients who achieved HemR, all obtained at least VGPR according to the ISA, and 90% by the IMWG criteria. This is despite only 15% of our patients receiving a triplet/quadruplet regimen based on daratumumab, which is currently the preferred first-line treatment for LC amyloidosis (8). More importantly, the advent of new therapies such as conjugated or bispecific antibodies may overcome these results shortly. On the other hand, according to the IMWG criteria, if patients do not have measurable disease at baseline, HemR can only be defined as a binary response. In contrast, the ISA criteria also propose an ungraded response when the difference between involved and uninvolved FLC is between 20 and 50 mg/L (Supplementary Tables S1, S2).

Taken together, all these data highlight that the actual graded response for MGRS could be ungraded (complete response/non-complete response/no response) or binary (complete response or non-response) (Figure 4).

Our work faces limitations, such as its small sample and retrospective nature. Given that our study is based on laboratory and histological data, the retrospective approach is unlikely to have influenced the results. The small sample is a consequence of the rarity of this disease, as reflected in similar registries (15–17). However, the small size and the differences between our cohorts may have influenced the assessment of the response. To the best of our knowledge, the largest report on MGRS since the updated definition of 2019 included 100 cases of MGRS-NA from 12 countries (9). It should be noted that some large-population studies included symptomatic MM, which are no longer considered MGRS cases (18). One strength of this study is that the same pathologist evaluated all kidney samples. Although data on the incidence of MGRS-NA are limited, the histomorphological spectrum of our study is consistent with the literature (15–17). However, cryoglobulinemia-associated glomerulonephritis was possibly underrepresented in our study due to the exclusion of 2 cases due to renal infiltration by lymphoma and the lack of serum FLC results at diagnosis.

In our study, only 37.5% of patients with MGRS-NA had measurable disease according to the IMWG consensus, 12.5% by SPEP, 15.6% by UPEP, and 31.2% by sFLC assay. Nonetheless, 19% of MGRS-NA patients had no available UPEP at diagnosis. Although it could be stated that the low percentage of measurable disease is due to an over-representation of PGNMID in our series, a retrospective registry reported a detectable M-protein by sPEP or uPEP in 48% of MGRS patients (15). Moreover, in an international study comparing MGRS-A and MGRS-NA, the median M-protein detected in MGRS-NA patients was ~ 0.057 g/dL, with no statistically significant differences from MGRS-A (9), which is consistent with our results.

It could be argued that the high recognition of measurable disease by the sFLC assay in our study is due to the fact that 24% of the MGRS-NA cases were due to LC renal deposition, such as MIDD and LCPT. However, recently published studies, including a meta-analysis of 877 individuals with MGRS, showed an even higher prevalence of these two entities (42–57%) among MGRS-NA (9, 15–17). On the other hand, a retrospective study found no statistically significant differences between the rate of MGRS-A and MGRS-NA patients with dFLC >100 mg/dL (16), and an international research identified that the amount of sFLC detected by sFLC assay was statistically significantly higher in MGRS-NA compared to MGRS-A (9). Comparing hematological and renal response across studies is complex due to the heterogeneity in assessment methods, type of MGRS measured, and follow-up time. However, the overall HemR and RenalR rates in our series are consistent with those previously described (7, 19, 20).

## Conclusion

Our findings indicate that, due to the disease’s low burden in MGRS-NA patients, HemR evaluation cannot rely on the MIg component identified in renal histology. Our study highlights potential alternatives to ease clinical practice. Importantly, the ISA response criteria enabled HemR evaluation in a substantially larger proportion of MGRS-NA patients than the IMWG criteria, suggesting a more comprehensive approach to response assessment. An ungraded response (complete or non-complete response vs. no response) may be optimal for MGRS-NA patients with measurable disease. In contrast, a binary response (complete response vs. non-response) could be appropriate for those with unmeasurable disease. For patients with undetectable disease, renal response remains the only feasible treatment assessment.

## Data Availability

The datasets presented in this article are not readily available because De-identified data utilized for this study are available from the corresponding author upon reasonable request. Requests to access the datasets should be directed to amalia.domingo@quironsalud.es.
