# Supplementary material for: Hematologic response assessment in monoclonal gammopathy of renal significance: critical appraisal of current criteria
Source: Front Med (Lausanne). 2026 Sep 8;13:1902202. doi: 10.3389/fmed.2026.1902202 (PMC13593509; doi:10.3389/fmed.2026.1902202)
Supplement: Supplementary file 1 [file Table_1.DOCX]

| **Standard IMWG response criteria** | |
| --- | --- |
| **Stringent complete response (sCR)** | Complete response as defined below plus normal FLC ratio and absence of clonal cells in bone marrow biopsy by immunohistochemistry. |
| **Complete response (CR)** | Negative immunofixation on the serum and urine and disappearance of any soft tissue plasmacytomas and <5% plasma cells in bone marrow aspirates. * |
| **Very good partial response (VGPR)** | Serum and urine M-protein detectable by immunofixation but not on electrophoresis or ≥90% reduction in serum M-protein plus urine M-protein level <100 mg per 24 h. ** |
| **Partial response (PR)** | ≥50% reduction of serum M-protein plus reduction in 24 h urinary M-protein by ≥90% or to <200 mg per 24 h.  If the serum and urine M-protein are unmeasurable, a ≥50% decrease in the difference between involved and uninvolved FLC levels is required in place of the M-protein criteria.  If serum and urine M-protein are unmeasurable, and serum-free light assay is also unmeasurable, ≥50% reduction in plasma cells is required in place of M-protein, provided baseline bone marrow plasma-cell percentage was ≥30%.  In addition to these criteria, if present at baseline, a ≥50% reduction in the size (SPD) of soft tissue plasmacytomas is also required. |
| **Minimal response (MiR)** | ≥25% but ≤49% reduction of serum M-protein and reduction in 24-h urine M-protein by 50–89%.  In addition to the above listed criteria, if present at baseline, a ≥50% reduction in the size (SPD) of soft tissue plasmacytomas is also required. |
| **Stable disease (SD)** | Not meeting criteria for complete response, very good partial response, partial response, minimal response, or progressive disease. |

**Supplementary Table 1. Standard IMWG response criteria.**

* When the only method to measure disease is by serum FLC levels: complete response can be defined as a normal FLC ratio of 0·26 to 1·65 in addition to the complete response criteria listed previously.

** When the only method to measure disease is by serum FLC levels: very good partial response requires a ≥90% decrease in the difference between involved and uninvolved FLC levels.

† If patients do not have measurable disease at baseline they can only be assessed for at least a complete response or progressive disease.
