# Supplementary material for: Hematologic response assessment in monoclonal gammopathy of renal significance: critical appraisal of current criteria
Source: Front Med (Lausanne). 2026 Sep 8;13:1902202. doi: 10.3389/fmed.2026.1902202 (PMC13593509; doi:10.3389/fmed.2026.1902202)
Supplement: Supplementary file 2 [file Table_2.DOCX]

| **Response categories**  **AL Amyloidosis** | **Original definitions**  **(ISA 2012)** | **Updated definitions**  **(Palladini et al. 2021)** |
| --- | --- | --- |
| **Complete response (CR)** | Both criteria must be met:   - Negative serum and urine immunofixation - Normal FLC ratio | Both criteria must be met:   - Absence of amyloidogenic light chains (either free and/or as part of a complete immunoglobulin) defined by negative immunofixation electrophoresis of both serum and urine - Either a FLC ratio within the reference range or the uninvolved FLC concentration is greater than involved FLC concentration with or without an abnormal FLC ratio |
| **Very good partial response**  **(VGPR)** | dFLC concentratio*n* < 40 mg/L | dFLC concentratio*n* < 40 mg/L |
| **Partial response (PR)** | dFLC decrease > 50% compared to baseline | dFLC decrease > 50% compared to baseline |
| **No response** | All other patients | All other patients |

**Supplementary Table 2. Validated hematologic response criteria for AL amyloidosis.**

† For patients with a dFLC value between 50 and 20 mg per liter, a hematologic response other than a complete response is reached when the dFLC value falls below 10 mg per liter.

FLC denotes free light chain, and dFLC denotes difference between amyloidogenic (involved) and non-amyloidogenic (uninvolved) free light chain concentrations.
